# Dynamics of Bacterial Communities and Resistomes Across Swine Waste Stabilization Ponds and Fertilized Soils

**DOI:** 10.1007/s00284-026-05026-6

**Published:** 2026-06-18

**Authors:** Oscar Victor Cardenas Alegria, Mariana Costa Torres, Gabriela Merker Breyer, Raquel Rebelatto, Camila Rosana Wuaden, Janaina Pastore, Mateus Lazzarotti, Rommel Thiago Juca Ramos, Marcio Dorn, Jalusa Deon Kich, Franciele Maboni Siqueira

**Affiliations:** 1 Laboratory of Bioinformatic and Genetic of Microorganisms, Center for High Performance Distributed Computing (CCAD), Federal University of Pará, Pará, Belém, 66075-110 Brazil; 2 Laboratório de Bacteriologia Veterinária (LaBacVet), Departamento de Patologia Veterinária, Universidade Federal do Rio Grande do Sul, Porto Alegre, Brazil; 3 Programa de Pós-Graduação em Ciências Veterinárias, Faculdade de Veterinária, Universidade Federal do Rio Grande do Sul, 9090 Bento Gonçalves Ave. 42704., Porto Alegre, 91540-000 Rio Grande do Sul Brazil; 4 Empresa Brasileira de Pesquisa Agropecuária - EMBRAPA Suínos e Aves, Concórdia, Brazil; 5 Laboratório de Bioinformática Estrutural e Biologia Computacional, Universidade Federal do Rio Grande do Sul, Porto Alegre, Brazil

## Abstract

The environmental dissemination of antimicrobial resistance (AMR) through livestock waste represents a growing concern for human, environmental, and animal health. This study investigated how swine waste stabilization ponds (WSPs), and subsequent manure application to agricultural soils, influence bacterial community structure, antimicrobial resistance genes (ARGs), and mobile genetic elements (MGEs). Using shotgun metagenomics, we analyzed 80 samples from 20 swine farms, including waste collected before and after WSP treatment and soils with and without a history of manure application. Distinct microbial profiles were observed between waste and soil environments. Waste samples were dominated by *Bacillota*, *Bacteroidota*, and *Pseudomonadota*, whereas soils were enriched in *Actinomycetota*, particularly *Streptomyces*. WSP significantly reduced microbial diversity and caused shifts toward stress-tolerant taxa, indicating selective pressures during the process. Manure-fertilized soils exhibited altered community composition and enrichment of clinically relevant ARGs, including the fluoroquinolone resistance gene *adeF*. Waste management practices influenced resistome composition, with treated waste showing increased relative abundance of macrolide resistance genes (*ermB* and *mefA*). In soils, ARG profiles were associated with distinct MGE patterns, suggesting environment-specific mechanisms of gene mobility. Phage-associated elements were more prevalent in waste samples, whereas transposons were more prominent in soils, where ARG-MGE co-occurrence patterns indicated potential for horizontal gene transfer. Overall, our findings demonstrate that WSP management and soil application of swine manure shape both microbial communities and resistome configurations. These results underscore the importance of integrating waste treatment strategies into AMR surveillance frameworks and support a One Health approach to mitigate its dissemination in agroecosystems.

**Supplementary Information:**

The online version contains supplementary material available at 10.1007/s00284-026-05026-6.

## Introduction

Global pig production has increased over the past 50 years, reaching approximately 120 million tons in 2018. Total pork consumption is estimated to increase by 13% in 2030 and 22% in 2050, compared to 2020 levels [1]. Thus, while a single pig can produce up to 6.4 kg of wet manure per day, large animal production facilities generate large quantities of animal feces, resulting in the production of 1.7 billion tons of feces annually worldwide [2]. The physicochemical and microbiological composition of pig manure from commercial farms exhibits substantial variability, influencing the selection and efficiency of treatment processes [3, 4]. The presence of different compounds, such as estrogens [5], heavy metals, antibiotics, and biocides, can adversely affect the ecosystem. Thus, the waste is treated and reused to achieve waste mitigation and the sustainable use of natural resources [6]. Swine manure treatment systems have proven effective in removing *Escherichia coli* [7] and in eliminating heavy metals through bioprocesses [8, 9]. Consequently, these treatment systems represent a valuable strategy for safeguarding environmental quality as well as animal and human health [10].

Waste treatment strategies encompass a range of approaches, including solid–liquid separation, aerobic treatment, anaerobic digestion, natural treatment systems, and integrated anaerobic–aerobic processes [11]. Among these, waste stabilization ponds (WSPs) are a widely adopted natural management system in livestock production, consisting of sequential ponds that operate in an open, continuous flow [12]. These treatments are designed to stabilize residues and facilitate their safe reuse as nutrient-rich fertilizers, supplying micronutrients and organic matter that enhance plant growth in agricultural soils [13]. Nevertheless, the application of animal manure as fertilizer carries microbiological risks, such as contamination with bacteria [9], antimicrobial resistance genes (ARGs), and mobile genetic elements (MGEs). Although evidence indicates that manure application can increase ARG abundance and reshape microbial communities, significant knowledge gaps persist regarding the fate of ARGs and MGEs during WSP treatment and their subsequent dissemination in soils amended with treated manure. In particular, it remains uncertain whether WSP processes reduce, maintain, or selectively enrich specific ARG classes, and how these dynamics influence ARG mobility once residues are applied to agricultural soils.

Within a One Health framework for antimicrobial resistance (AMR) surveillance, environmental metagenomics serves as a powerful tool to identify the diversity and composition of microorganisms, antimicrobial resistance genes (ARGs), mobile genetic elements (MGEs), and heavy metal resistance genes [14–17]. Brazil ranks among the world’s largest producers and exporters of animal protein, with swine farming representing a major income source in rural areas—particularly in the southern region—where it generates substantial economic benefits [18]. Against this backdrop, the present study seeks to characterize bacterial communities, ARGs, and MGEs that may be transferred to the environment through fertilizers derived from waste stabilization ponds (WSPs), given their relevance to environmental, human, and animal health. Therefore, this study aimed to characterize the diversity and composition of bacterial communities, ARGs, and MGEs in samples of WSPs and soils fertilized with organic material from WSPs.

## Materials and Methods

### Sample Collection

A total of 20 swine farms using WSP from southern Brazil – Rio Grande do Sul (*n* = 14) and Santa Catarina (*n* = 6) – were included in this study, comprising nursery (*n* = 10), and grow-to-finish (*n* = 10) production systems (Supplementary Table 1). All farms were visited in November 2023. From each farm, four sample points were selected: (1) the raw swine manure at the entry of the management systems (non-treated waste); (2) the manure from WSPs (treated waste); (3) non-fertilized soils, including native and farmstead areas (non-fertilized soil); and (4) farmland areas with organic fertilizer obtained from the digestion system (fertilized soils).

Briefly, the raw swine manure and manure samples were collected in sterile 2,000 mL containers for homogenization, and 50 mL was transferred to a sterile tube and refrigerated until sample processing. Soil sampling was performed by drilling to a depth of 30–40 cm, and approximately 2 kg of soil was collected in a sterile container. The soil was homogenized, and 10 g was transferred to a sterile tube and transported to the laboratory under refrigerated conditions.

### Metagenomic DNA Extraction and Sequencing

Liquids from WSP were centrifuged at 138 x g for 1 min to remove debris; then, 15 mL of the supernatant was centrifuged again at 16,000 x g for 20 min. The pellet was resuspended in 300 µL of ultra-pure water. For the soil samples, 250 mg of homogenized soil was directly used for metagenomic DNA extraction. DNA isolations were performed using the DNeasy^®^ PowerSoil^®^ kit (Qiagen, Hilden, Germany) according to the manufacturer’s protocol. The quality and quantity of the isolated DNAs were determined by NanoDrop (ThermoScientific, Waltham, MA, USA) and Qubit 2.0 Fluorometer (Thermo Scientific, Waltham, MA, USA), respectively.

Metagenomic DNAs that match the quality and quantity parameters (260/230 and 260/280 rations range of 2.0-2.2 and at least 100 ng/ul of DNA) were subject to shotgun sequencing. Libraries were sequenced on the NovaSeq 6000 platform (Illumina, San Diego, CA, USA), with a paired-end (2 × 150 bp) strategy.

### Metataxonomic Characterization

Raw reads quality was assessed using FastqC 0.11.9 [19], and low-quality sequences (Phred < 30) were removed using Trimmomatic v.3 [20]. For taxonomic identification, filtered reads were processed using Kraken2 2.1.2 [21] with a database for identification of bacteria, archaea, and viruses. Pavian 1.2.1 [22] was employed to generate a comparative matrix across all waste and soil samples, which was used in the MicrobiomeAnalyst 2.0 [23], enabling the transformation of microbial abundances using Trimmed Mean of M-values (TMM). Alpha (Shannon, Simpson, and Observed) and beta diversity indices were calculated (ANOSIM test).

### Determination of Antimicrobial Resistance Genes and Mobile Elements

The ARGs were identified by processing the clean reads using CARD-RGI 4.0.2 [24]. Then the files were processed to extract the abundance information, followed by normalization using the formula: $\:Cij\:=\:1000\:*\:\surd\:(Rij/Tij)$, described previously by Inda-Díaz et al. [25]. Where, *Cij* is the normalized abundance, *Rij* is the number of aligned reads in a gene class “*i*”, in a sample “*j*”, and “*Tj*” is the total number of reads obtained in a sample “*j*”. Likewise, genes with ≥ 80% coverage were selected, followed by the selection of the characteristic’s “mechanism”, “drug class”, and “ARO_gene”. A minimum gene coverage threshold of 80% was applied to reduce alignments derived from short conserved domains while retaining biologically meaningful gene matches. Lower thresholds may inflate false-positive detections by including partial or homologous fragments, whereas more stringent thresholds may exclude incompletely assembled genes, increasing false-negative rates.

The reads were assembled using the MegaHit 1.2.9 [26] to generate contigs to identify MGEs. The quality of the contigs were evaluated using the Quast 5.2.0 [27]. MobileGO Beatrix 1.6 [28] was used to identify the different MGEs with high identity (> 90%; e-value < 10^− 5^). The identified MGEs were manually classified into plasmids, transposons, and phages using the Uniprot [29] and National Center for Biotechnology Information (www.ncbi.nlm.nih.gov) databases.

### Statistical Analysis

Normality was assessed using the Shapiro-Wilk test. Subsequently, comparisons of diversity median among the different samples were conducted using Wilcoxon or Mann Whitney tests (p-value < 0.05). In the comparative analysis of gene abundances, these were initially log-transformed, and subsequently pairwise differences were calculated between the corresponding sample groups. For PCA analysis, the data obtained from the diversity and abundance of ARG and MGE genes were normalized using Z-scores, followed by a correlation analysis prior to performing the PCA. These analyses were performed using the PAST 4.17 [30] and R 4.4.1 (www.r-project.org). Infographics were created using the seaborn package [31], the Orange DataMine program version 3.36.1 [32], and the Flourish website [33].

## Results

The metagenomic sequencing yield was higher in the soil samples, with an average of 36,087,890 reads in the fertilized soil samples, followed by 36,012,730 reads in non-fertilized soil samples. In waste samples, the yield was higher in digested waste samples, with 30,299,130 reads, while raw swine manure samples had an average of 28,451,570 reads. The sequencing and assembly metrics were shown in Supplementary Table 1. On average, 13 to 39.2% of the reads were classified into different organisms, of which 11 to 32.4% were bacteria.

### Diversity Indexes are Related to the type of Sample

The bacterial composition across the samples showed similar alpha diversity rates among the analyzed groups when evaluated using Observed and Simpson indices (Supplementary Table 2). However, lower values were observed in the Shannon index for the manure samples (Mann-Whitney; z = 2.799; *p* = 0.005) (Supplementary Table 2). The beta diversity analysis revealed significant differences between the bacterial composition of waste and soil samples (ANOSIM; *R* = 0.69176; *p* < 0.001) (Supplementary Fig. 1). On the other hand, when the samples from each group (origin) were analyzed, no variations were observed between the two types of waste samples, non-treated and treated (ANOSIM; *R* = 0.147; *p* < 0.002), as well as between fertilized and non-organic fertilized soil samples (ANOSIM; *R* = 0.434; *p* < 0.001).

### The Bacterial Composition Changes Across the Waste Treatment Process

The composition of the bacterial communities at the phylum level differed between waste and soil samples. A higher abundance of the phyla *Bacillota*,* Bacteroidota*, and *Pseudomonadota* was observed in the waste samples, while *Actinomycetota* was present in lower proportions. In the digested waste samples, there was a higher abundance of *Spirochaetota*. However, similarities were observed in the phylum composition of the soil samples. Interestingly, the phyla *Acidobacteriota* and *Pseudomonadota* were more prominent in the non-fertilized soil samples, while *Actinomycetota* stood out in fertilized soil samples.

**Fig. 1:**
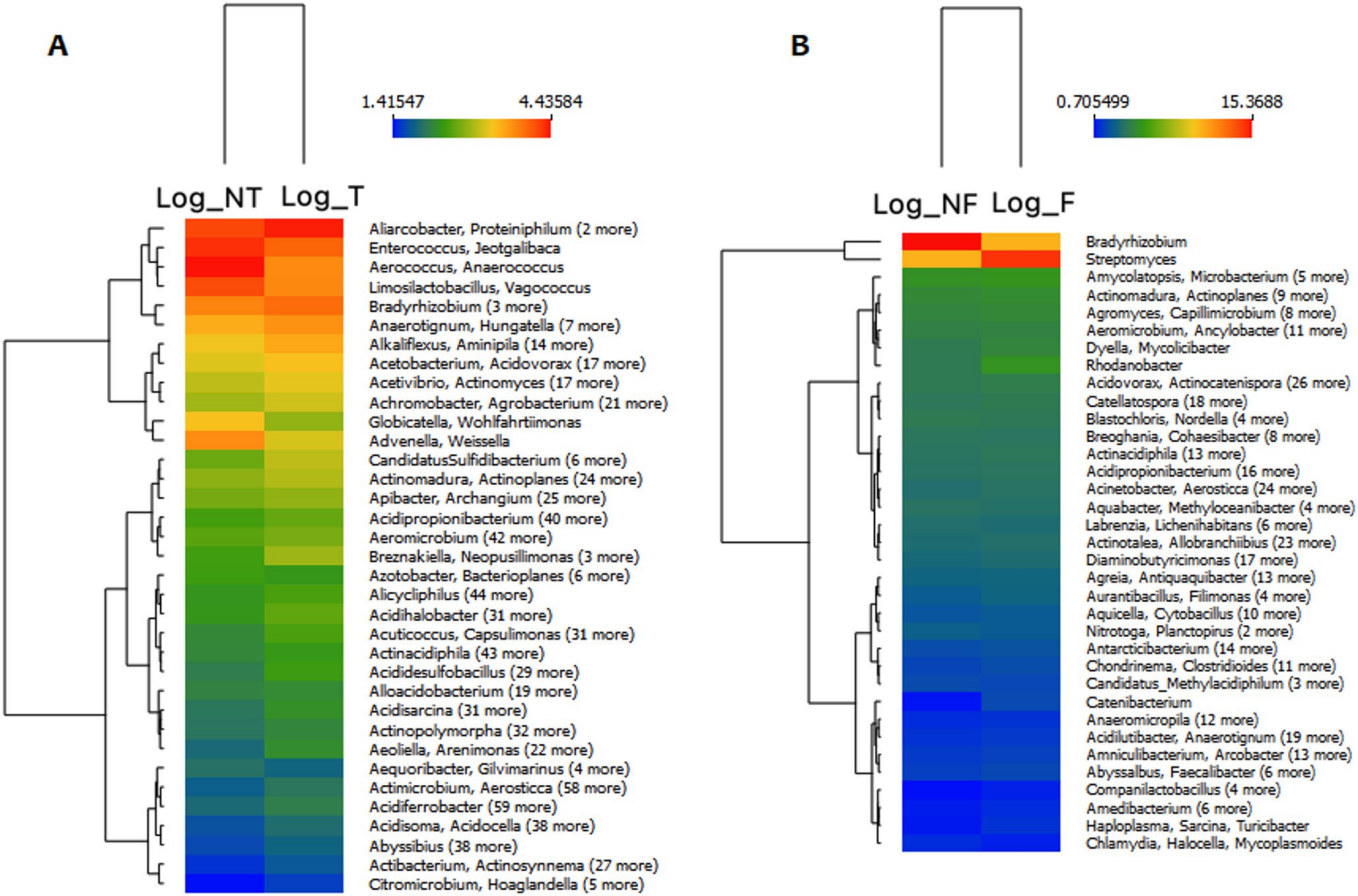
Heatmap of the bacterial domain composition, at the taxonomic level, of the genus from the different collection points. Groupings were performed based on abundance values and treated with logarithms “log” for points **A**. of treated (Log_T) and untreated (Log_NT) waste and **B**. of non-fertilized (Log_NF) and fertilized (Log_F) soils

At the genus level, bacterial communities that showed significant differences among the samples were selected (Wilcoxon test; *p* < 0.05) and grouped in a heatmap according to their mean abundances. In the raw swine manure and manure samples, *Aliarcobacter*,* Proteiniphilum*,* Streptomyces*, and *Anaerococcus* showed high abundance levels, with greater abundance in the manure samples compared to raw swine manure. In addition, the genera *Advenella*,* Weisella*,* Globicatella*, and *Wohlfahrtiimonas* were also highly abundant in these samples (Fig. 1). In the case of soil samples, *Bradyrhizobium* stood out in the non-fertilized samples, whereas *Streptomyces* showed high abundance in soils with manure as fertilizer (Fig. 1).

A deeper analysis of the bacterial community from swine WSPs and soils fertilized with their content was based on the bacterial species level. Among the most abundant bacterial species identified in the waste samples, in the digested samples there was a decrease in the species *Segatella copri*,* Denitrificimonas caeni*, and *Prevotella herbatica*, and an increase in *Sphaerochaeta associata* and *Vescimonas fastidiosa* species (Supplementary Fig. 2A). On the other hand, in the soil samples, we observed a decrease in the species *Bradyrhizobium erythtophei*,* Rhodoplanes* sp. Z2 YC6860, and *Rhodopseudomonas palustris*, and an increase in *Gemmatirosa kalamazoonensis* and *Capillimicrobium parvum* abundances (Supplementary Fig. 2B).

### Antimicrobial Resistance Genes are Maintained in Fertilized Soils

The putative ARGs identified in WSP samples had an average abundance of 40.45 ± 7.416 and 37.85 ± 5.752 ARGs from non-treated and treated samples, respectively. Fisher’s alpha genetic diversity index values were 17.184 ± 4.357 in non-treated samples and 15.052 ± 2.936 in treated samples. In the case of soil samples, an average abundance of 83.05 ± 36.427 ARGs was detected in non-fertilized soil samples, in contrast to 86.1 ± 28.358 ARGs in fertilized soil samples. The values of Fisher’s alpha diversity indices were 31.447 ± 11.33 for non-fertilized samples, and 29.675 ± 6.685 for fertilized samples.

**Fig. 2:**
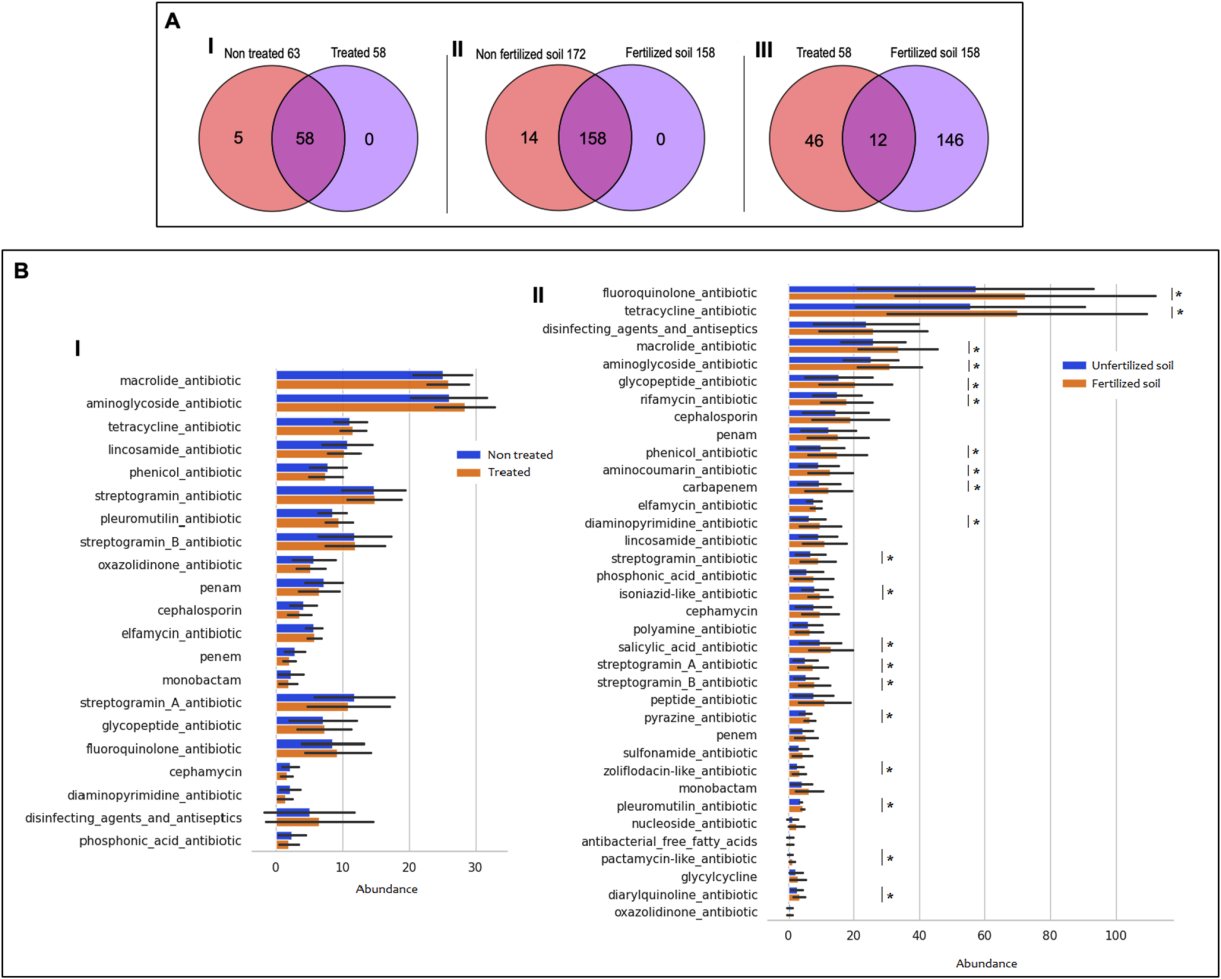
Diversity of antimicrobial resistance genes. **A**: Vem Euler diagram of the presence of antimicrobial resistance genes identified in samples of I: waste samples, II: soil samples, and III: slurry (digested waste) and soil fertilezed with the slurry. **B**: Antimicrobial classes targets of the resistance genes identified in the samples of I: waste samples and II: soil samples (*Mann–Whitney test; *p* > 0.05)

The presence of different genes in the samples showed a higher number of ARGs in the soil samples compared to the waste samples (Fig. 2A). Raw swine manure samples had the highest number of exclusive ARGs (Fig. 2A, panel I), while in soil samples, the non-fertilized soils showed the highest values (Fig. 2A, panel II). Comparisons of digested waste and fertilized soils with the manure showed the sharing of 12 ARGs, in addition to a greater number of ARGs that were exclusive to fertilized soil (Fig. 2A, panel III). The identified ARGs decreased after waste management, and similarly, a smaller proportion of these genes was observed in the soil when it was applied as organic fertilizer (12 ARGs).

When the relative abundance of antimicrobial classes was analyzed (Fig. 2), we observed that macrolide and aminoglycoside classes were the most abundant in WSP samples (Fig. 2B, panel I). Nevertheless, in the soil samples significative differences between fertilized and non-fertilized soils were identified for fluoroquinolones (Mann–Whitney test; z = 2.799; *p* = 0.005), tetracyclines (Mann–Whitney test; z = 2.394; *p* = 0.017), macrolides, and aminoglycosides (Mann–Whitney test; z = 2.583; *p* = 0.009), among others (Fig. 2B, panel II).

To assess the most common ARGs in the different samples, we analyzed the differential abundance of the identified ARGs. Mutations in the *16 S-DNA* and *23 S*-*DNA* genes exhibited the highest average abundances of ARGs identified in the waste samples (Supplementary Fig. 3A). In the soil samples, the most prominent ARGs was *adeF*, followed by *qacG* (Supplementary Fig. 3B).

**Fig. 3:**
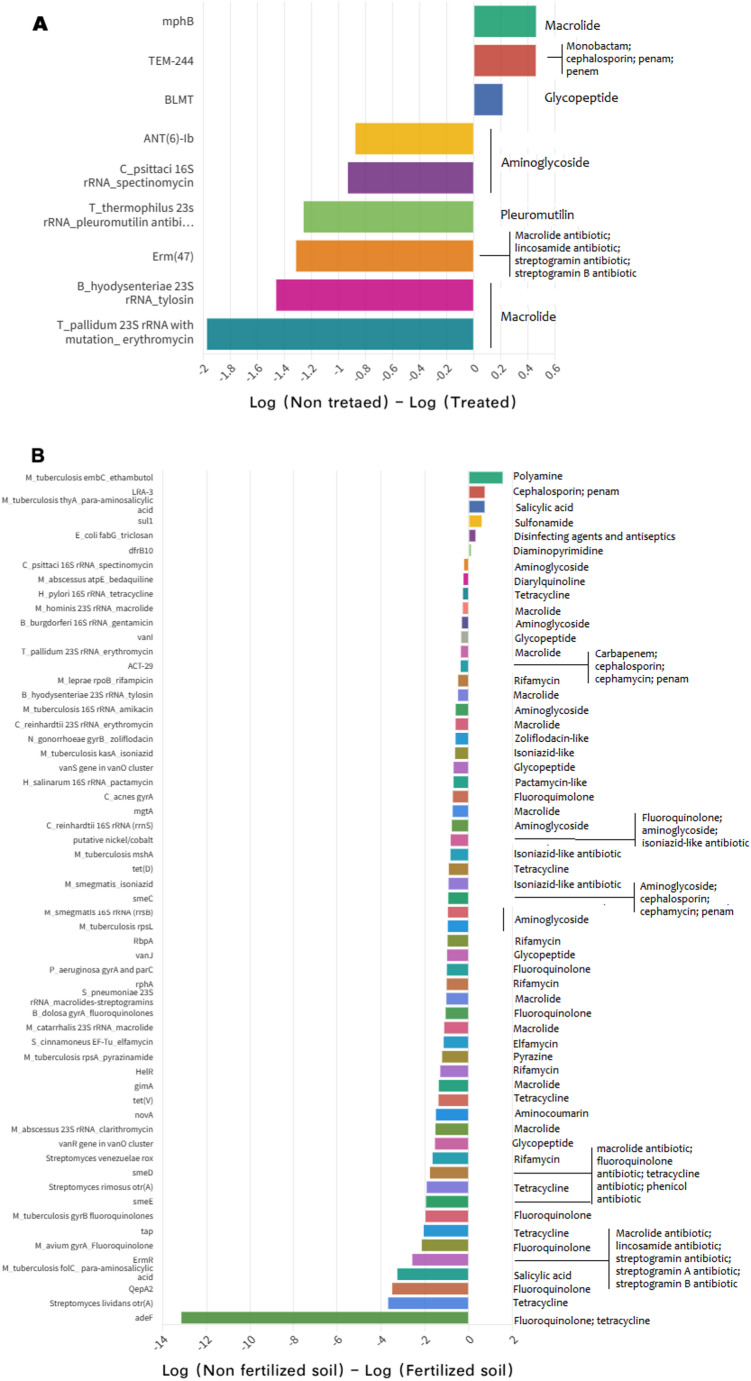
Differential abundance of antimicrobial resistance genes. **A**: Comparisons between samples from raw swine manure (non-treated) and slurry (treated). **B**: Comparisons between soil fertilizer with slurry and control soil (without slurry fertilization). These genes were subtracted from the means of the samples from each group. Wilcoxon test; *p* < 0.05

By comparing the mean of the ARG abundance in waste samples from both, non-treated and treated collections, nine ARGs showed significant differences (*p* < 0.05) in their abundance (Fig. 3A), which indicates an increase in the abundance of genes involved in resistance, with emphasis on macrolide resistance ARGs after waste management.

In the fertilized and non-fertilized soil samples, the comparisons led to the selection of 59 ARGs (*p* < 0.05). Most of these genes presented negative values (Fig. 3B), which suggests an increase in these ARGs in soil samples after the land application of manure as fertilizer, especially the *adeF* gene, which is involved in resistance to fluoroquinolones and tetracyclines.

The analysis of the resistance mechanisms of the ARGs identified in the different samples showed that, in the WSP samples, “target site alteration”, followed by “efflux pumps” were the most prominent mechanisms (Supplementary Fig. 4A). In contrast, the soil samples showed a higher prevalence of “efflux pumps”, followed by the mechanism of “target site alteration” and “antibiotic inactivation” (Supplementary Fig. 4B).

### Mobile Genetic Elements are Present in the Entire Process of Manure Treatment and Organic Soil Fertilization

The MGEs in the raw swine manure samples exhibited an average abundance of 266.8 ± 97,833 genes, while the digested samples showed an average abundance of 214.4 ± 0,526. Fisher’s alpha diversity index values were 42,094 ± 17,524 and 34,921 ± 8,281 for the raw swine manure and WSP digested samples, respectively. On the other hand, in the soil samples, the average abundance of MGEs was lower than in waste samples, with 25.3 ± 6.096 genes in non-fertilized soil and 36.55 ± 18.693 genes in fertilized soil, with significant differences between them (Mann-Whitney; z = 2.111; *p* = 0.034). When the Fisher’s alpha diversity index was applied the values were 5.618 ± 1.304 and 8.443 ± 3.348 in non-fertilized and fertilized soil samples, respectively (Mann-Whitney; z = 3.422; *p* = 0.001).

**Fig. 4:**
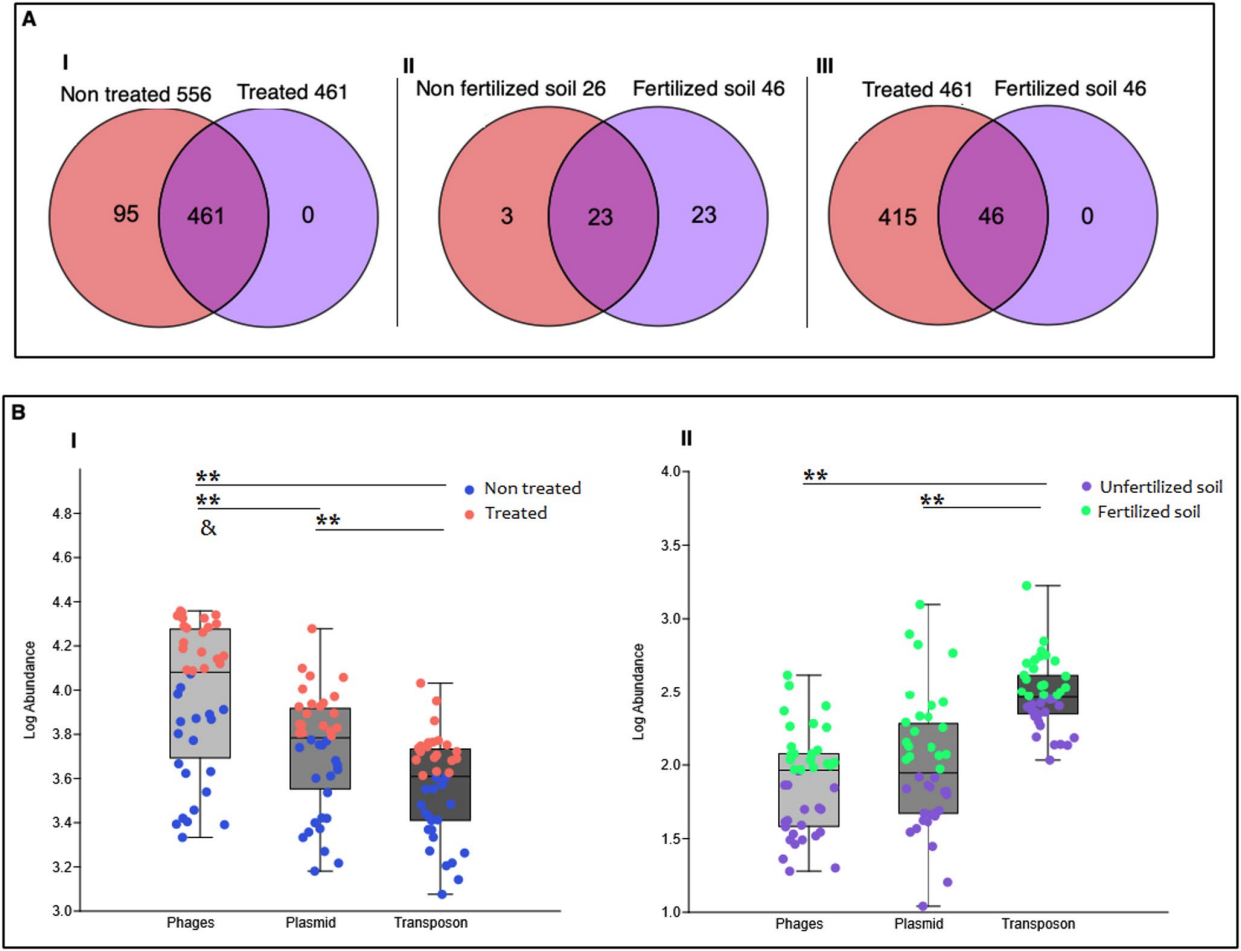
Diversity of mobile genetic elements (MGEs) in the analyzed samples. **A**: Vem Euler diagram of the MGEs in samples I: waste samples, II: soil samples, and III: slurry (digested waste) and soil fertilized with the slurry. **B**: Abundance of MGE genes in the different samples analyzed I: waste samples and II: soil samples (&: *p* < 0.05; **: *p* < 0.001)

The analysis of genes involved in horizontal gene transfer (HGT) revealed a higher abundance of MGEs in waste samples compared to soil samples (Fig. 4A). In waste samples, 461 MGEs were shared between swine manure and digested groups (Fig. 4A, panel I). In soil samples, 23 MGEs were shared between fertilized and non-fertilized soils, with three MGEs unique to non-fertilized samples (Fig. 4A, panel II). In contrast, treated waste and fertilized soil samples shared only 46 MGEs, while the majority were unique to treated waste (415) and none were unique to fertilized soil (Fig. 4A, panel III). Noteworthy, no MGEs were found exclusively in the fertilized soil. These results suggest a closer similarity between the raw swine manure and digested waste microbial communities regarding MGE composition, whereas fertilized soil samples harbor distinct MGE profiles, likely influenced by soil management practices.

The MGE distribution varied among sample types, with higher gene abundance observed particularly in manure samples, followed by organic fertilized soil samples (Fig. 4B). In waste samples, phages were the most abundant MGE category, followed by plasmids and transposons, with significant differences among them (Mann-Whitney test; *p* < 0.001; Fig. 3B). Conversely, in soil samples, transposons showed the highest abundance, followed by plasmids and phages (Fig. 4B), with statistically significant differences observed between transposons and both phages and plasmids (Mann-Whitney test; *p* < 0.001). Additionally, a significant difference in phage abundance was detected between raw swine manure and digested waste samples (Mann-Whitney test; *z* = 2.448; *p* = 0.014).

In the analysis of MGE abundance across different samples, waste samples exhibited a higher abundance of MGEs than soil samples. Based on the average abundances, distinct gene clusters were observed in samples from WPS, including those associated with transposons, phages, and plasmid elements. The average gene abundances in soil samples revealed higher values in samples that were not fertilized with swine manure. The genes with the highest abundances were those involved in transposons. Notably, the presence of the EF0125 gene, related to a transposon element, was exclusively present in non-fertilized soil samples.

**Fig. 5:**
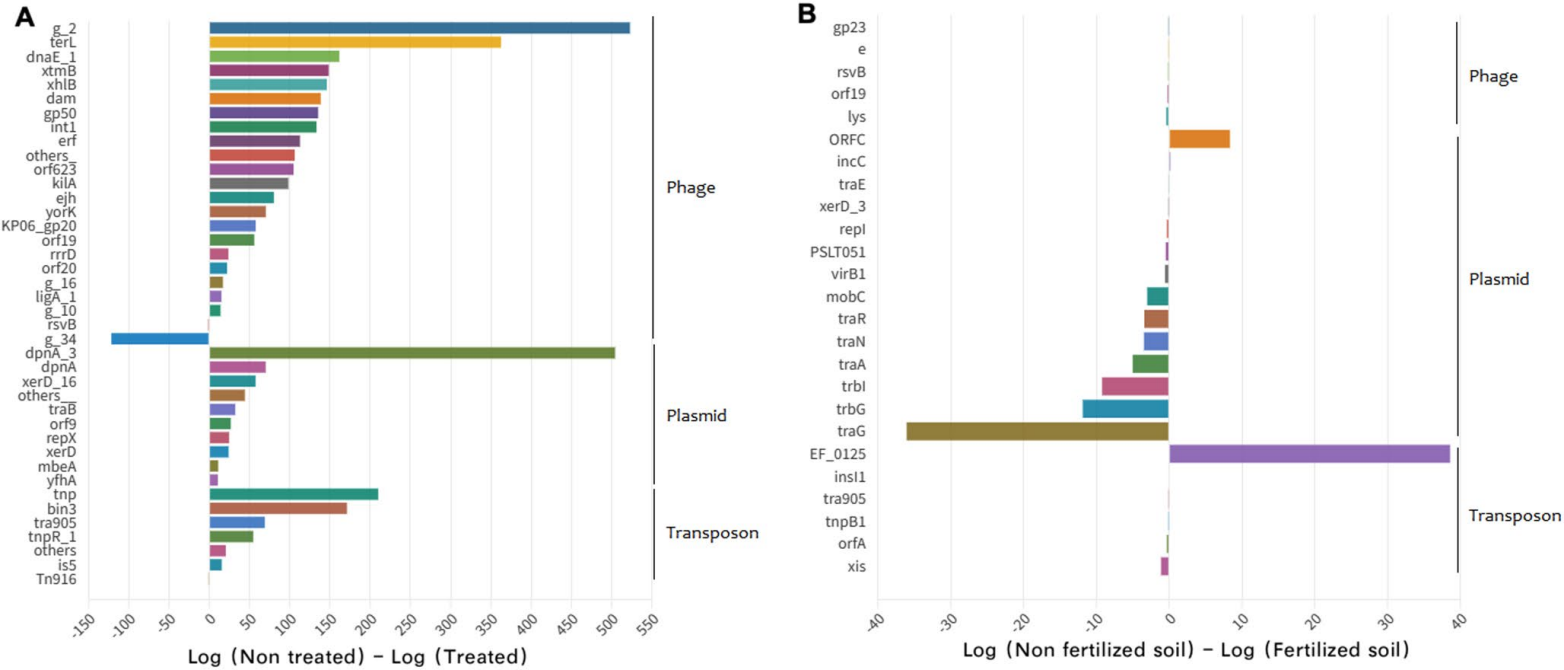
Differential abundance of genes related to mobile genetic elements (MGE) in the samples. **A**. Differential abundance between raw swine manure (non-treated) and slurry (treated) samples. Positive values indicate greater abundance in the non-treated waste samples. **B**. MGEs differential abundance between non-fertilized and fertilized soil samples. Positive values indicate greater abundance in the non-fertilized soil samples. Negative values indicate greater relative abundance in fertilized soil. Wilcoxon test; (*p* < 0.05)

According to the differential abundance analysis of MGE genes in the waste samples, a significant difference (*p* < 0.05) in average gene abundance was detected at the end of the waste management process (Fig. 5A). The lower averages observed in the treated waste samples suggest a reduction in these genes following the waste management system.

In contrast, the differential abundance analysis of MGE genes in soil samples showed more subtle effects, with higher gene abundance observed in fertilized soils (Fig. 5B). Notably, an increase in the abundance of certain transposon genes in fertilized soil (Fig. 5B) was observed, which may suggest enhanced genetic mobility in soils fertilized with swine waste.

### Antimicrobial Resistance Dissemination through Organic Fertilized Soils

**Fig. 6:**
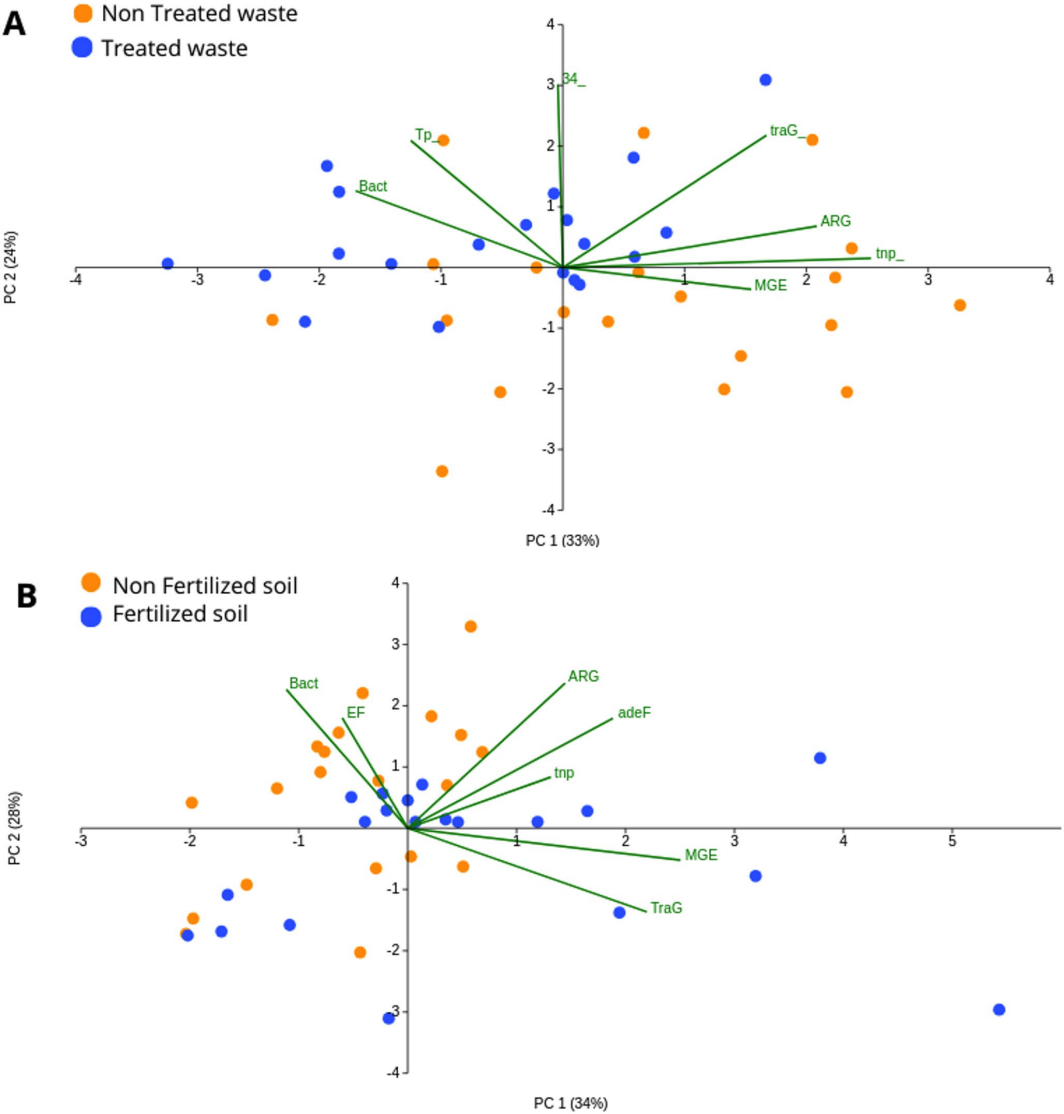
Principal Component Analysis of alpha diversity indices of bacteria, antimicrobial resistance genes, and mobile genetic elements. **A**. Raw swine manure (non-treated - “orange dot”) and slurry (treated - “blue dot”). **B**. Non-fertilized (“orange dot”) and slurry-fertilized (“blue dot”) soil samples. Green vectors represent the alpha diversity indexes for each gene group (bacteria “Bact”, Resistance genes “ARG”, such as genes resistance of effacing (*EF*), fluoroquinolone (adeF) and erythromycin (Tp); and genes of mobile elements “MGE”) the conjugal transfer mating (*TraG*), RNA polymerase sigma GP34 (*34*) and IS1182 transposase (*tnp*) with the highest contribution to variation between groups

To investigate the potential dissemination of antimicrobial resistance (AMR) across environmental compartments, we conducted a principal component analysis (PCA) to explore relationships among bacterial alpha diversity indices, ARGs, and MGEs in swine waste and soil samples (Fig. 6). From the associations observed in the waste samples, it was possible to show that the values of the alpha diversity indices of mobilome genes (MGEs) and resistance (ARGs) are associated, especially in untreated samples. It was also observed that the values of the alpha diversity index of bacteria and the diversity index of MGEs were inversely proportional (Fig. 6A).

In the soil samples, the associations were negative between the values of the bacterial alpha diversity index and the diversity of MGE genes. Likewise, the diversity indices of MGE associations between the diversity indices of MGE genes were associated with conjugation genes, and these were relevant in fertilized soils. The bacterial alpha diversity indices were associated with the abundance of the elfamycin resistance gene, mainly in non-fertilized soils (Fig. 6B).

Statistically significant correlations were found between ARG diversity and the presence of the transposase gene *tnp* in waste samples (Spearman’s *ρ* = 0.795, *p* = 0.027), and between MGE diversity and the genes *dpnA3* (*ρ* = 0.808, *p* = 0.016), *phage2* (*ρ* = 0.791, *p* = 0.033), *terL* (*ρ* = 0.746, *p* = 0.0002), and *bin3* (*ρ* = 0.76, *p* = 0.009) in treated samples. In raw swine manure samples, MGE diversity correlated with the gene *bln* (*ρ* = 0.657, *p* = 0.001), as shown in Supplementary Fig. 5A. In soils, the presence of the gene *adeF* was strongly associated with ARG diversity in both fertilized (*ρ* = 0.844, *p* = 0.02) and non-fertilized soils (*ρ* = 0.8, *p* = 0.02). Additionally, MGE diversity in fertilized soils correlated significantly with the gene *traG* (*ρ* = 0.795, *p* = 0.02), as illustrated in Fig. 6B and Supplementary Fig. 5B.

## Discussion

The analysis of bacterial composition revealed that the soil samples have higher alpha diversity values than waste samples. These results are indicative of the greater complexity of soil, as well as the difference in sample composition observed in beta diversity analysis [2]. The diversity of microbial communities in both waste and manure samples can be closely related to the host’s intestinal microbiome. Diversity changes are related to the management that occurs during the processing of these samples, mainly due to the variation of physical and chemical components and the modification of organic matter, affecting the substrates for bacterial metabolism [34], the influence of chemical components is relevant to the structure of microbial communities, which could also influence the diversity of the samples collected, data not obtained.

Furthermore, we also observed the occurrence of bacteria usually associated with the swine mucosa microbiota in the raw swine manure samples, such as *Prevotella*, *Lactobacillus*, and *Bifidobacterium*, which have a great abundance in the mucosa and are associated with health benefits, and *Campylobacter*, *Clostridium*, *Veillonella*, and *Helicobacter*, which are potentially harmful or associated with intestinal dysbiosis [35]. Also, in the digested samples, *Betaproteobacteria* were prominent, highlighting their strong capacity of biodegradation of organic matter in swine waste, as *Burkholderiales* are involved in fermentative processes [36].

The genus *Streptomyces* was present in both types of analyzed samples, waste and soil, being more prominent in soil samples. These bacteria have the capacity for carbohydrate degradation, fermentation and, above all, production of compounds that help plant development, such as phytohormones [37, 38]. Additionally, the production of substances with biocontrol capabilities against fungi and other bacteria that are pathogenic to plants is described for *Streptomyces* [37, 38].

Among the bacterial species identified in the waste samples (Supplementary Fig. 2A), those that are part of the intestinal microbiota of pigs were highlighted, such as *Segatella copri*, which can adapt to the environment and has been described as involved in swine pathogenic processes [39]. Interestingly, *Segatella copri* genomes have a significant presence of ARGs and MGEs [39]. Another species highlighted was *Denitrificimonas caeni*, which could be involved in the waste treatment as it can reduce nitrite to nitrogen [40]. *Sphaerochaeta associata*, which exhibits a high ability to adapt to extreme environments [41, 42], was identified as the bacterial species with the greatest abundance after the WSP system (Supplementary Fig. 2A). Noteworthy, bacterial species from both fertilized and non-fertilized soils were predominantly involved in plant nutrient processing and plant adaptation to environmental conditions (Supplementary Fig. 2A), such as species from the genera *Bradyrhizobium*, *Rhodoplanes*, and *Rhodopseudomonas* [43, 44].

Considering the ARGs, the diversity was higher in the soil samples compared to the WSP samples. These results indicate that the WSP system can influence the loss of both bacterial diversity and genetic diversity, as previously stated by He et al. [45]. Previous studies have highlighted the use of swine compost as fertilizer, because the ARG transfer is lower in swine compost when compared to composts from different origins [46]. Noteworthy, ARGs found exclusively in organic fertilized soils may be introduced either from human activity or from native community bacteria [47, 48]. In the environment, some antimicrobial drugs can remain persistent and exert strong selective pressure for the maintenance of bacterial resistance. For instance, fluoroquinolone and tetracycline antibiotics are difficult to degrade in the environment [49]. Moreover, the dominance of tetracycline resistance genes has been frequently found in human and animal feces, which may be related to the historical selection for tetracyclines commonly used throughout the world [50].

At the gene level, many *adeF* homologous sequences have been detected in various bacteria from manure samples [51]. Additionally, genes conferring tetracycline resistance—*tetA*,* tetB*,* tetC*,* tetG*,* tetM*, and *tetX*—have been reported [52, 53], likely due to the co-occurrence of ARGs in fertilized soils of swine origin. However, the increasing abundance of ARGs in soil should be carefully considered during fertilization, as these genes can be transferred to plants [48, 54].

In animals, due to changes in the intestinal absorption of antimicrobials, the presence of antibiotic target alteration mechanisms in the feces was observed [55], which could explain the abundance of this resistance mechanism found in the WSP samples from this present study. However, soil samples highlight a higher distribution of efflux pumps, which are mainly a response to environmental stress [56]. These efflux pumps are involved in multidrug resistance [57] and could be an important factor in the presence of opportunistic pathogenic bacteria.

In addition to the ARGs, the presence of MGEs may play a crucial role in facilitating the transmission of ARGs between bacteria in different ecosystems, contributing to the adaptation of both the host and coexisting organisms [58]. MGEs were found throughout the treatment process and were identified near ARGs such as plasmids, transposons and integrons. Understanding such relationships can guide the selection of appropriate control measures, emphasizing the prudent use of antimicrobials and effective waste disposal [59]. The co-localization of ARGs and MGEs suggests the potential for HGT; however, we did not perform structural validation and the active mobility or transfer rates cannot be confirmed.

Previous studies have reported a higher diversity of MGEs in the intestinal microbiota, suggesting an enhanced capacity for HGT [51]. These MGEs are subsequently excreted and may disseminate into wastewater and soil environments. Notably, the conjugation-mediated transfer of antibiotic resistance genes in soil is dependent on soil composition [60], with suboptimal conditions leading to a reduction in both gene transfer and MGE diversity [61]. Therefore, the lower MGE diversity observed in our soil samples may be associated with specific soil components.

In raw swine manure samples, the notably high abundance of phage components may reflect the influence of physical and chemical variations, including substrate composition, which can shape microbial community structure by selecting bacteria with adaptive metabolic capabilities. Phages play a central role in modulating these bacterial communities through predation, generalized transduction, and the dissemination of accessory genes, including those conferring antibiotic resistance [62]. However, further virome-specific analyses are necessary to confirm the identity of these viral elements, as their abundance may be overestimated due to limitations in current classification pipelines.

Furthermore, the presence of integron systems in these environments likely contributes to increased genome plasticity, enhancing bacterial adaptability to environmental pressures, such as those imposed by manure treatment processes [63]. In soil samples, the elevated abundance of transposable elements—often associated with plasmids carrying ARGs – may be linked to the presence of selective agents with antimicrobial activity in the soil. These compounds can induce gene duplication and promote HGT of resistance determinants via MGEs [64, 65].

According to previous studies, manure application in soils enhances ARG diversity and abundance, largely due to organic matter enrichment [17, 66]. Long-term manure storage contributes to the enrichment of MGEs, including transposons and insertion sequences, thereby increasing the potential for ARG dissemination. Elements such as *tnpA* and *IS91* have been implicated in HGT among soil bacteria [67–69]. These findings underscore the importance of continuous monitoring of ARG and MGE diversity and abundance in soils receiving manure amendments to inform effective mitigation strategies and promote sustainable agricultural practices [66]. Overall, interpretations should be made cautiously due to limitations, including the cross-sectional design, the absence of antibiotic residue measurements, and the lack of physicochemical soil and waste metadata.

## Conclusion

This study revealed that although swine WSP systems reduce the abundance of ARGs and MGEs, they do not eliminate them entirely. The continued presence of ARGs, such as those associated with macrolides, tetracyclines, and fluoroquinolones, and the co-localization with MGEs such as plasmids and transposons in digested waste and fertilized soils underscores the potential for HGT. The increase in ARG abundance and diversity in fertilized soils suggests that the application of digested waste contributes to the propagation of environmental AMR. These findings emphasize the importance of robust monitoring systems, improved waste management protocols, and sustainable land practices to mitigate the risks associated with the environmental spread of ARGs. From a practical perspective, our findings support the need for routine surveillance of ARGs and MGEs in waste treatment systems and agricultural soils receiving manure. Integrating physicochemical monitoring, antibiotic residue quantification, and standardized resistome assessments could improve risk evaluation.

## Supplementary Information

Below is the link to the electronic supplementary material.

Supplementary Material 1

## Data Availability

The data supporting the findings of this study are available at the National Center for Biotechnology Information under BioProject number PRJNA1321884 (BioSamples SAMN51227481-SAMN51227652).
