# Supplementary material for: Dynamics of Bacterial Communities and Resistomes Across Swine Waste Stabilization Ponds and Fertilized Soils
Source: Curr Microbiol. 2026 Jun 18;83(8):436. doi: 10.1007/s00284-026-05026-6 (PMC13279407; doi:10.1007/s00284-026-05026-6)
Supplement: Supplementary file 1 — Supplementary Material 1 [file 284_2026_5026_MOESM1_ESM.docx]

Dynamics of bacterial communities and resistomes across swine waste stabilization ponds and fertilized soils

Oscar Victor Cardenas Alegria^1^, Mariana Costa Torres^2,3^, Gabriela Merker Breyer^2,3^,Raquel Rebelatto^4^, Camila Rosana Wuaden^4^, Janaina Pastore^4^, Mateus Lazzarotti^4^, Rommel Thiago Juca Ramos^1^, Marcio Dorn^5^, Jalusa Deon Kich^4^, Franciele Maboni Siqueira^2,3*^

^1^Instituto de Ciências Biológicas, Universidade Federal do Pará, Belém, Pará, Brazil

^2^Laboratório de Bacteriologia Veterinária (LaBacVet), Universidade Federal do Rio Grande do Sul, Departamento de Patologia Veterinária, Porto Alegre, Brazil

^3^Programa de Pós-Graduação em Ciências Veterinárias, Faculdade de Veterinária, Universidade Federal do Rio Grande do Sul, Porto Alegre, Brazil

^4^Empresa Brasileira de Pesquisa Agropecuária - EMBRAPA Suínos e Aves, Concórdia, Brazil

^5^Laboratório de Bioinformática Estrutural e Biologia Computacional, Universidade Federal do Rio Grande do Sul, Porto Alegre, Brazil

*Corresponding Author: franciele.siqueira@ufrgs.br

Tel. +55 51 3308 6165

9090 Bento Gonçalves Ave. 42704. Porto Alegre, Rio Grande do Sul, Brazil. Zip code: 91540-000.

Supplementary Fig. 1 – Beta diversity analysis among the waste and soil samples


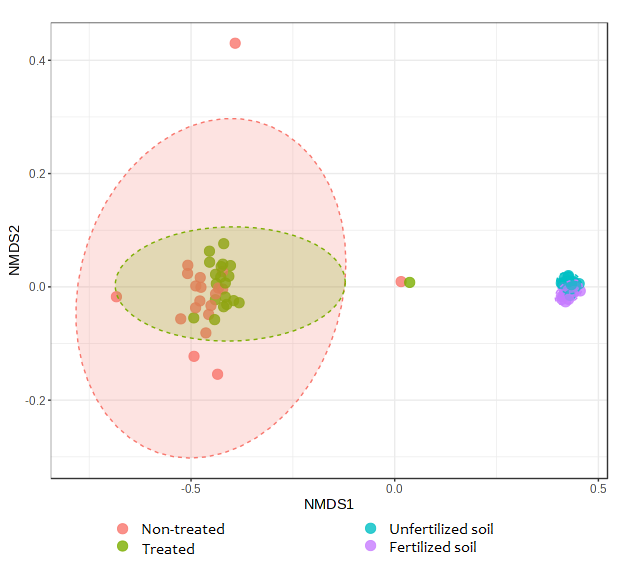


Supplementary Fig. 2 – Relative abundance of the bacterial community at the species level


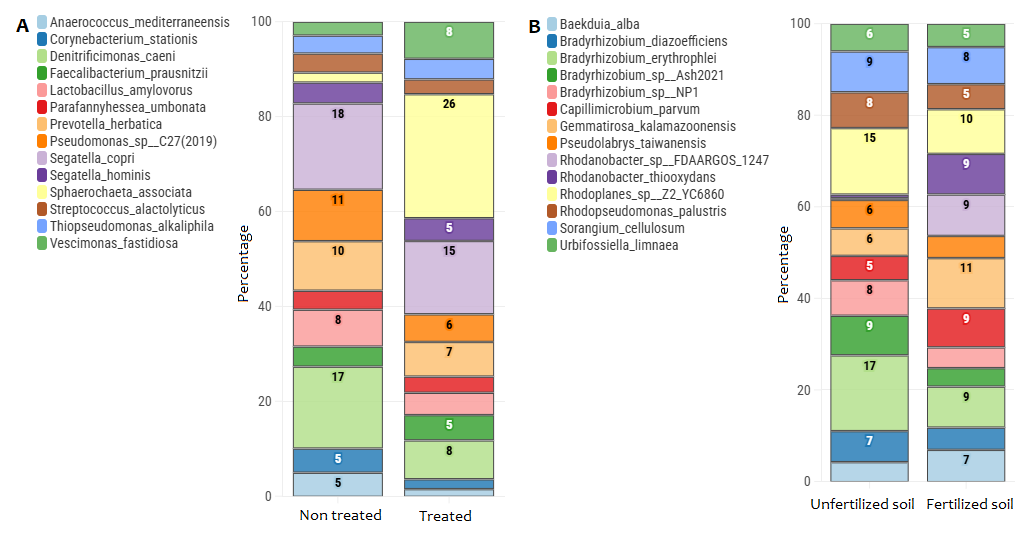


Percentage distribution of the most abundant species in the samples. A: Raw swine manure (Non Treated) and slurry (Treated). B: non-organic fertilized (Non-fertilized) and organic fertilized (Fertilized) soils.

Supplementary Fig. 3 – Clustering of the mean abundance of antimicrobial resistance genes identified


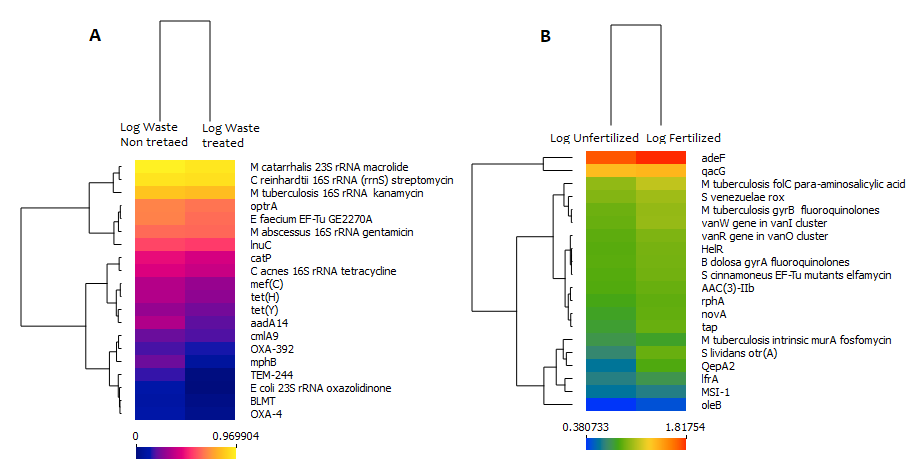


A: Raw swine manure (Non treated) and slurry (treated). B: non-organic fertilized (Unfertlized) and organic fertilized (Fertilized) soils.

Supplementary Fig. 4 - Resistance mechanisms conferred by antimicrobial resistance genes


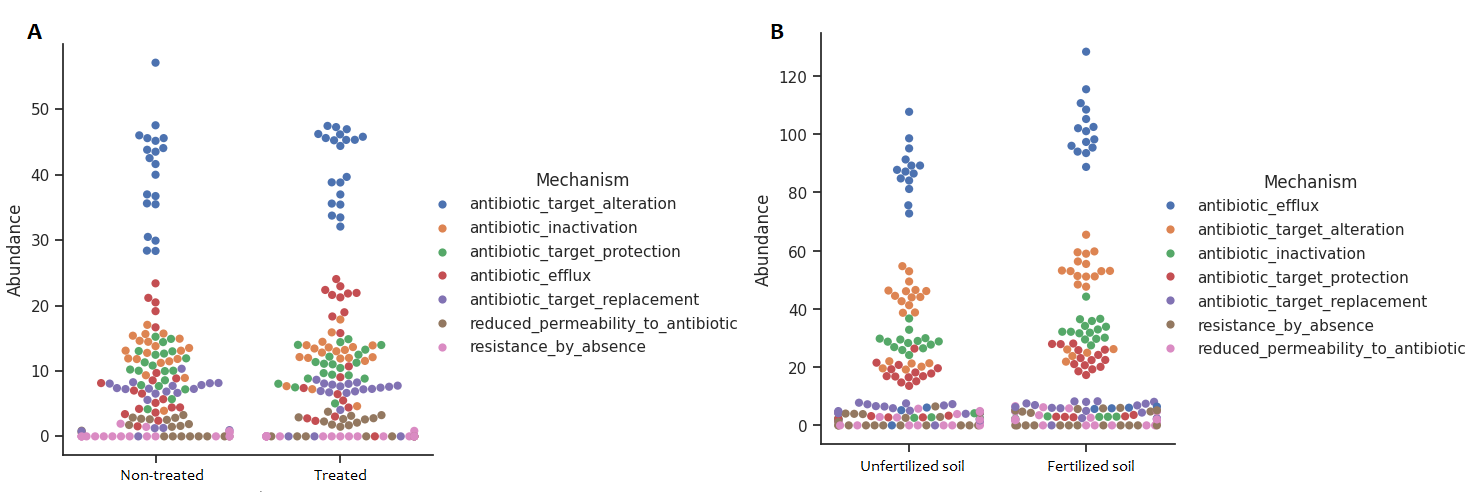


A: Raw swine manure (Non treated) and slurry (Treated). B: non-organic fertilized (Non-fertilized) and organic fertilized (Fertilized) soils.

Supplementary Fig. 5 – Spearman’s correlation between the diversity of antimicrobial resistance genes and mobile genetic elements


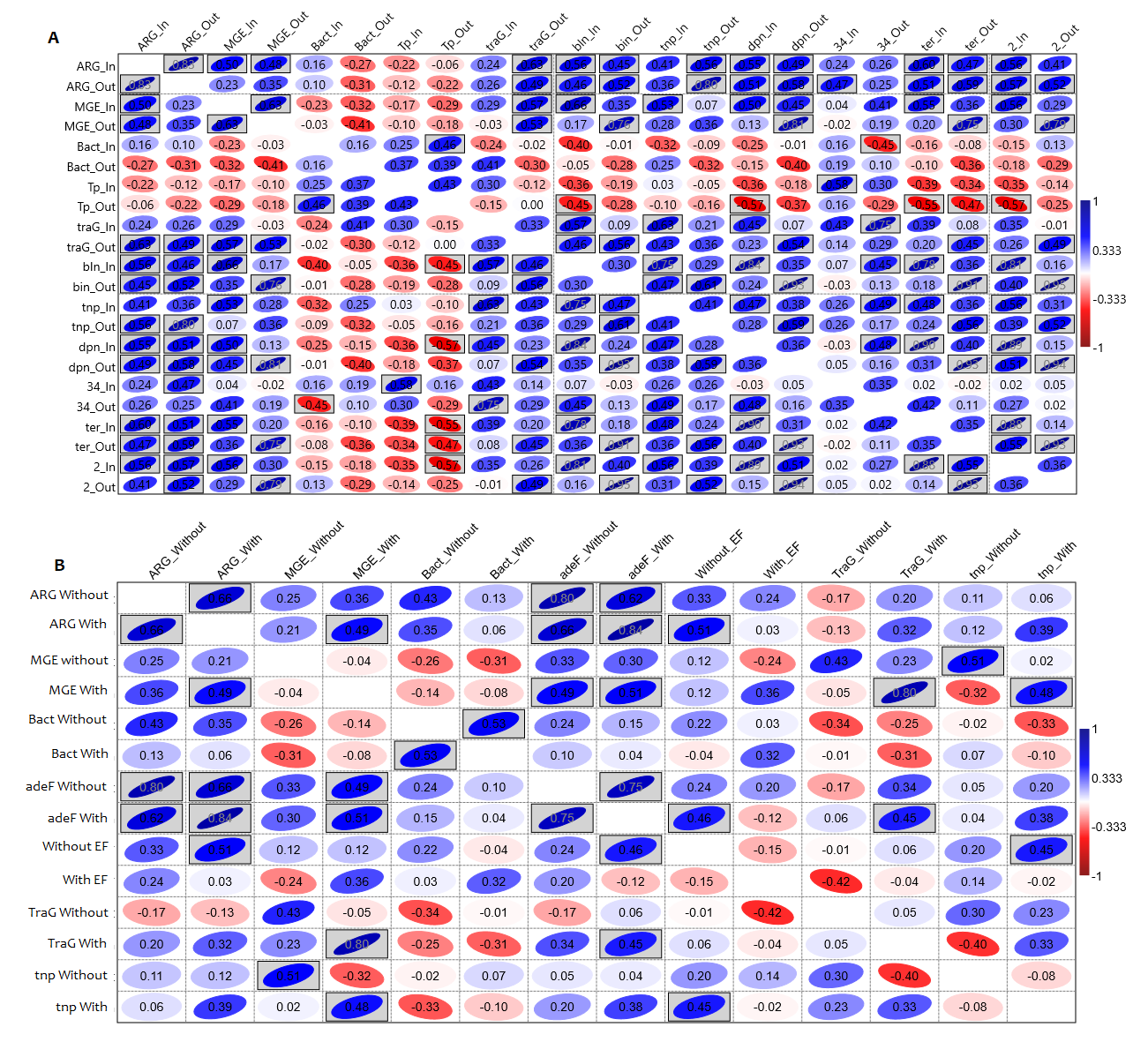


A: Raw swine manure (non-treated - “In”) and slurry (treated - “Out”). B: Non-fertilized (“Without”) and slurry-fertilized (“With”) soil samples.

Supplementary Table 1. Sequencing performance and assembly data income of waste and soil samples

| Nº | State | Samples | Type of production | Size | Number of raw reads (after filtering) | Contigs size | N50 |
| --- | --- | --- | --- | --- | --- | --- | --- |
| 1 | SC | WSP non-treated | Nursery | 9.1G | 26,420,249 | 444M | 1378 |
| 2 | SC | WSP treated | Nursery | 9.2G | 26,710,167 | 607M | 1471 |
| 3 | SC | Non-fertilized soil | Nursery | 12G | 32,466,715 | 1,2G | 822 |
| 4 | SC | Fertilized soil | Nursery | 11G | 30,208,108 | 712M | 701 |
| 5 | RS | WSP non-treated | Nursery | 7.1G | 20,493,402 | 577M | 1450 |
| 6 | RS | WSP treated | Nursery | 9.6G | 27,680,352 | 687M | 1490 |
| 7 | RS | Non-fertilized soil | Nursery | 8.3G | 23,929,523 | 530M | 748 |
| 8 | RS | Fertilized soil | Nursery | 12G | 32,628,543 | 946M | 805 |
| 9 | RS | WSP non-treated | Nursery | 10G | 28,952,252 | 637M | 1469 |
| 10 | RS | WSP treated | Nursery | 11G | 29,533,899 | 766M | 1368 |
| 11 | RS | Non-fertilized soil | Nursery | 12G | 34,294,418 | 801M | 715 |
| 12 | RS | Fertilized soil | Nursery | 12G | 32,138,901 | 1,2G | 937 |
| 13 | RS | WSP non-treated | Nursery | 8.5G | 24,549,798 | 650M | 1301 |
| 14 | RS | WSP treated | Nursery | 9.5G | 27,441,547 | 720M | 1261 |
| 15 | RS | Non-fertilized soil | Nursery | 12G | 32,309,568 | 699M | 721 |
| 16 | RS | Fertilized soil | Nursery | 14G | 38,543,324 | 1,3G | 877 |
| 17 | RS | WSP non-treated | Nursery | 11G | 29,273,314 | 562M | 1400 |
| 18 | RS | WSP treated | Nursery | 9.6G | 27,960,271 | 672M | 1508 |
| 19 | RS | Non-fertilized soil | Nursery | 12G | 33,451,214 | 946M | 747 |
| 20 | RS | Fertilized soil | Nursery | 11G | 31,232,018 | 792M | 744 |
| 21 | RS | WSP non-treated | Nursery | 9.7G | 28,072,710 | 746M | 1348 |
| 22 | RS | WSP treated | Nursery | 9.9G | 28,633,896 | 888M | 1319 |
| 23 | RS | Non-fertilized soil | Nursery | 11G | 31,779,439 | 1,1G | 810 |
| 24 | RS | Fertilized soil | Nursery | 12G | 32,981,154 | 795M | 716 |
| 25 | RS | WSP non-treated | Nursery | 16G | 45,379,595 | 1,2G | 1278 |
| 26 | RS | WSP treated | Nursery | 15G | 43,395,069 | 1,2G | 1304 |
| 27 | RS | Non-fertilized soil | Nursery | 16G | 46,156,729 | 1,3G | 736 |
| 28 | RS | Fertilized soil | Nursery | 18G | 49,697,795 | 1,6G | 802 |
| 29 | RS | WSP non-treated | Nursery | 17G | 46,693,495 | 1,3G | 1388 |
| 30 | RS | WSP treated | Nursery | 15G | 41,635,168 | 1,2G | 1342 |
| 31 | RS | Non-fertilized soil | Nursery | 18G | 51,033,325 | 1,2G | 733 |
| 32 | RS | Fertilized soil | Nursery | 20G | 55,541,456 | 1,4G | 730 |
| 33 | RS | WSP non-treated | Nursery | 14G | 40,031,610 | 1,1G | 1350 |
| 34 | RS | WSP treated | Nursery | 16G | 46,573,678 | 905M | 1390 |
| 35 | RS | Non-fertilized soil | Nursery | 23G | 64,823,864 | 2G | 747 |
| 36 | RS | Fertilized soil | Nursery | 9.6G | 27,855,496 | 891M | 811 |
| 37 | RS | WSP non-treated | Nursery | 3.7G | 10,578,628 | 262M | 1443 |
| 38 | RS | WSP treated | Nursery | 4.9G | 14,026,145 | 387M | 1414 |
| 39 | RS | Non-fertilized soil | Nursery | 4.5G | 13,009,488 | 315M | 759 |
| 40 | RS | Fertilized soil | Growing-to-finishing | 6.6G | 18,970,564 | 436M | 777 |
| 41 | SC | WSP non-treated | Growing-to-finishing | 6.0G | 17,390,625 | 400M | 1146 |
| 42 | SC | WSP treated | Growing-to-finishing | 6.3G | 18,091,412 | 336M | 1237 |
| 43 | SC | Non-fertilized soil | Growing-to-finishing | 6.2G | 17,904,098 | 351M | 700 |
| 44 | SC | Fertilized soil | Growing-to-finishing | 6.9G | 19,927,695 | 494M | 708 |
| 45 | SC | WSP non-treated | Growing-to-finishing | 4.3G | 12,480,477 | 509M | 1158 |
| 46 | SC | WSP treated | Growing-to-finishing | 11G | 30,978,813 | 833M | 1368 |
| 47 | SC | Non-fertilized soil | Growing-to-finishing | 13G | 36,796,844 | 1006M | 756 |
| 48 | SC | Fertilized soil | Growing-to-finishing | 12G | 34,506,503 | 764M | 679 |
| 49 | SC | WSP non-treated | Growing-to-finishing | 12G | 33,200,880 | 1,2G | 1214 |
| 50 | SC | WSP treated | Growing-to-finishing | 13G | 38,025,716 | 1003M | 1488 |
| 51 | SC | Non-fertilized soil | Growing-to-finishing | 12G | 32,689,589 | 618M | 671 |
| 52 | SC | Fertilized soil | Growing-to-finishing | 14G | 38,811,281 | 1,2G | 729 |
| 53 | SC | WSP non-treated | Growing-to-finishing | 9.9G | 28,811,868 | 1G | 1166 |
| 54 | SC | WSP treated | Growing-to-finishing | 8.9G | 25,975,702 | 798G | 1182 |
| 55 | SC | Non-fertilized soil | Growing-to-finishing | 15G | 41,683,428 | 1,5G | 757 |
| 56 | SC | Fertilized soil | Growing-to-finishing | 14G | 40,661,014 | 1,4G | 764 |
| 57 | SC | WSP non-treated | Growing-to-finishing | 12G | 33,411,620 | 825M | 1267 |
| 58 | SC | WSP treated | Growing-to-finishing | 12G | 34,324,253 | 838M | 1303 |
| 59 | SC | Non-fertilized soil | Growing-to-finishing | 14G | 39,692,571 | 1,2G | 743 |
| 60 | SC | Fertilized soil | Growing-to-finishing | 12G | 33,388,642 | 937M | 747 |
| 61 | RS | WSP non-treated | Growing-to-finishing | 9.5G | 27,684,096 | 813M | 1159 |
| 62 | RS | WSP treated | Growing-to-finishing | 8.9G | 25,913,806 | 701M | 1194 |
| 63 | RS | Non-fertilized soil | Growing-to-finishing | 15G | 41,642,840 | 1005M | 698 |
| 64 | RS | Fertilized soil | Growing-to-finishing | 13G | 36,336,805 | 794M | 683 |
| 65 | RS | WSP non-treated | Growing-to-finishing | 11G | 31,082,604 | 893M | 1251 |
| 66 | RS | WSP treated | Growing-to-finishing | 9.3G | 27,081,965 | 770M | 1140 |
| 67 | RS | Non-fertilized soil | Growing-to-finishing | 13G | 36,649,526 | 1,4G | 854 |
| 68 | RS | Fertilized soil | Growing-to-finishing | 12G | 33,195,956 | 871M | 803 |
| 69 | RS | WSP non-treated | Growing-to-finishing | 9.9G | 28,658,184 | 574M | 1220 |
| 70 | RS | WSP treated | Growing-to-finishing | 11G | 30,565,802 | 941M | 1146 |
| 71 | RS | Non-fertilized soil | Growing-to-finishing | 12G | 32,800,504 | 909M | 737 |
| 72 | RS | Fertilized soil | Growing-to-finishing | 14G | 39,866,657 | 1,4G | 771 |
| 73 | RS | WSP non-treated | Growing-to-finishing | 11G | 31,265,813 | 954M | 1221 |
| 74 | RS | WSP treated | Growing-to-finishing | 11G | 31,449,774 | 835M | 1231 |
| 75 | RS | Non-fertilized soil | Growing-to-finishing | 15G | 42,132,954 | 1,4G | 739 |
| 76 | RS | Fertilized soil | Growing-to-finishing | 14G | 39,030,362 | 1,2G | 785 |
| 77 | RS | WSP non-treated | Growing-to-finishing | 8.4G | 24,600,252 | 791M | 1075 |
| 78 | RS | WSP treated | Growing-to-finishing | 11G | 29,985,185 | 965M | 1120 |
| 79 | RS | Non-fertilized soil | Growing-to-finishing | 13G | 36,511,073 | 1,1G | 751 |
| 80 | RS | Fertilized soil | Growing-to-finishing | 19G | 54,732,378 | 1,6G | 694 |

WSP: waste stabilization pond; SC: Santa Catarina State; RS: Rio Grande do Sul State.

Supplementary Table 2. Values ​​of alpha diversity indices in the different analyzed samples

|  | Observed | Shannon | Simpson |
| --- | --- | --- | --- |
| Waste non-treated | 7433,05 ±33,449 | 6,611 ±0,688* | 0,983 ±0,025 |
| Waste treated | 7446,85 ±11,481 | 7,084 ±0,325* | 0,989 ±0,0166 |
| Fertilized soil | 7419,7 ±36,336 | 7,720 ±0,098 | 0,998 ±0,001 |
| Non-fertilized soil | 7392,6 ±56,521 | 7,737 ±0,086 | 0,999 ±0,0002 |

*p-value< 0.05
